# Self-poisoning suicide deaths in people with bipolar disorder: characterizing a subgroup and identifying treatment patterns

**DOI:** 10.1186/s40345-017-0081-9

**Published:** 2017-04-27

**Authors:** Ayal Schaffer, Lauren M. Weinstock, Mark Sinyor, Catherine Reis, Benjamin I. Goldstein, Lakshmi N. Yatham, Anthony J. Levitt

**Affiliations:** 1 Mood and Anxiety Disorders Program, Department of Psychiatry, Sunnybrook Health Sciences Centre, 2075 Bayview Avenue, Room FG 52, Toronto, ON M4N 3M5 Canada; 2 Department of Psychiatry, University of Toronto, Toronto, Canada; 3 Department of Psychiatry and Human Behavior, Brown University, Providence, RI USA; 4 Department of Psychiatry, Sunnybrook Health Sciences Centre, Toronto, Canada; 5 Centre for Youth Bipolar Disorder, Department of Psychiatry, Sunnybrook Health Sciences Centre, Toronto, Canada; 6 Departments of Psychiatry and Pharmacology, University of Toronto, Toronto, Canada; 7 Department of Psychiatry, University of British Columbia, Vancouver, Canada

**Keywords:** Bipolar disorder, Suicide death, Self-poisoning, Overdose, Lethal medications

## Abstract

**Objective:**

To characterize self-poisoning suicide deaths in BD compared to other suicide decedents.

**Methods:**

Extracted coroner data from all suicide deaths (*n* = 3319) in Toronto, Canada from 1998 to 2012. Analyses of demographics, clinical history, recent stressors, and suicide details were conducted in 5 subgroups of suicide decedents: BD self-poisoning, BD other methods, non-BD self-poisoning, non-BD other methods, and unipolar depression self-poisoning. Toxicology results for lethal and present substances were also compared between BD and non-BD self-poisoning subgroups as well as between BD and unipolar depression self-poisoning subgroups.

**Results:**

Among BD suicide decedents, self-poisoning was significantly associated with female sex, past suicide attempts, and comorbid substance abuse. In both the BD and non-BD self-poisoning groups, opioids were the most common class of lethal medication. For both groups, benzodiazepines and antidepressants were the most common medications present at time of death, and in 23% of the BD group, an antidepressant was present without a mood stabilizer or antipsychotic. Only 31% of the BD group had any mood stabilizer present, with carbamazepine being most common. No antidepressant, mood stabilizer, or antipsychotic was present in 15.5% of the BD group. Relative to unipolar depression self-poisoning group, the BD self-poisoning group evidenced higher proportion of previous suicide attempt(s) and psychiatry/ER visits in the previous week.

**Conclusion:**

People with BD who die by suicide via self-poisoning comprise a distinct but understudied group. The predominant absence of guideline-concordant pharmacologic care comprises a crucial target for future policy and knowledge translation efforts.

## Background

Suicide is one of the most common causes of death for people with bipolar disorder (BD) (Angst et al. 2005; Høyer et al. 2000; Ösby et al. 2001; Pompili et al. 2013a). Persons with BD account for up to 10% of all suicide deaths (Chen et al. 2009; Clements et al. 2013; Ilgen et al. 2010; Karch et al. 2006; Schaffer et al. 2014; Takizawa 2012), and have an estimated standardized mortality ratio of 10–30 compared to the general population (Crump et al. 2013; Harris and Barraclough 1997; Kessing et al. 2005; Pompili et al. 2013a).

Suicide by self-poisoning is a prevalent cause of death globally. Self-poisoning accounts for approximately a quarter of all suicides in England with females and the young being particularly vulnerable to this method of suicide (Camidge et al. 2003; Kapur et al. 2005). Methods of suicide deaths in BD vary across studies and countries, but self-poisoning deaths are consistently found to be the first or second most common method (Chen et al. 2009; Dennehy et al. 2011; Gos et al. 2009; Hunt et al. 2006; Isomets and Henriksson 1994; Keks et al. 2009; Ösby et al. 2001; Rihmer et al. 1990; Schaffer et al. 2014). Self-poisoning accounted for 25–30% of deaths in the three largest studies that reported on BD suicide methods (Chen et al. 2009; Hunt et al. 2006; Ösby et al. 2001) and 17–53% in the smaller studies (Angst et al. 2005; Clements et al. 2013; Crump et al. 2013; Harris and Barraclough 1997; Høyer et al. 2000; Ilgen et al. 2010; Karch et al. 2006; Kessing et al. 2005; Pompili et al. 2013a; Schaffer et al. 2014; Takizawa 2012); however, there is a paucity of data specifically focusing on characterizing this subgroup of suicide deaths in BD. Furthermore, BD also presents as a vulnerable psychiatric population to self-poisoning with elevated prevalence among BD decedents relative to non-BD decedents (33.5 vs. 17.4%; Schaffer et al. 2014) and schizophrenia decedents (34.9 vs. 17.4%; Sinyor et al. 2015). Little is known about who is more likely to die from this method, which substances are ingested around the time of death, and which of these specific substances were ultimately lethal. In a small Finnish study examining 11 people with BD who died by self-poisoning suicide, lethal ingestion of an antipsychotic occurred in 45% of cases, followed by 18% with each of a tricyclic antidepressant, lithium, or combination of benzodiazepines (Isomets and Henriksson 1994).

There is a modest amount of data on treatments received near the time of death. In an Australian review of 35 BD suicide deaths by any method, a clinical panel assessed only 34% of decedents as having received adequate therapeutic interventions (pharmacological and psychological) prior to death (Keks et al. 2009). For instance, while 43% of patient had taken lithium within 4 weeks of deaths, only 11% had adequate therapeutic lithium levels.

A similar pattern of underutilization of adequately dosed pharmacotherapy was found in a Finnish sample of 31 BD suicides (Isomets and Henriksson 1994). We are not aware of any prior studies that extensively report on the presence of specific classes of medications based on toxicology at the time of death, a more robust approach that ensures that the compound was in fact being ingested. With self-poisoning deaths being common and a method of suicide that physicians as prescribers have some control over, it is important to better characterize this BD subgroup in order to understand who is more likely to die from this method, and what medications or other substances were taken around the time of death. The objective of this study was to examine these data in a large sample of suicide decedents with BD and comparison groups.

## Methods

### Data source

The Office of the Chief Coroner for Ontario (OCC) investigates all suicide deaths in Toronto, Canada. As part of a large study of OCC data on all suicides in the City of Toronto from 1998 to 2012 (*n* = 3319 suicide deaths) (Schaffer et al. 2014; Sinyor et al. 2012, 2014), we examined 5 subgroups: (1) BD suicide by self-poisoning; (2) BD suicide by other methods; (3) non-BD suicide by self-poisoning; (4) non-BD, non-self-poisoning; (5) unipolar depression by self-poisoning.

OCC charts include a coroner’s investigation report, pathology report, toxicology report, and collateral information gathered from interviews with family or others, physician/clinical records, police reports, and copies of suicide notes. OCC data are not available for approximately 2 years after the death while investigations are completed. Deaths were classified as suicides based on a standard of a high degree of probability. We did not include deaths that were considered indeterminate with respect to suicide as the cause.

A standardized data extraction procedure was used, collecting data on: (1) demographics: age, sex, marital status, living circumstances; (2) clinical variables: the presence of a BD diagnosis, unipolar depression, substance abuse history (including alcohol, drugs or both), past suicide attempts, reported contact with psychiatric or emergency services in the week prior to death, comorbid medical condition; (3) recent stressors: employment/financial, interpersonal stressor, medical/health, police/legal, bereavement; and (4) details of suicide: method, presence of a suicide note and location of suicide.

Demographic data and details of the suicide were available in 99% of coroner charts. Clinical and stressor variables were only included in the coroner charts if they were present. All clinical and stressor variables were therefore considered estimates, with possible underreporting.

For the purpose of this study, any person with BD who died by intentional self-poisoning, as a single or combination method of suicide, was included in the BD self-poisoning suicide group. All other BD suicide deaths were included in a non-self-poisoning suicide group. As additional comparison groups, we also included self-poisoning and non-self-poisoning suicide deaths in people without BD as well as a unipolar depression self-poisoning group.

A detailed toxicology report was available for most deaths by self-poisoning (93.4% of BD group and 85.1% of the non-BD group). The OCC primarily conducts toxicology tests in cases where self-poisoning is suspected, therefore no toxicology data are available for the group of BD suicides by other methods. Toxicology reports include information on the level and distribution of all substances detected in the blood or urine, and the approximate time since death. The pathologist uses this information to determine whether the substance was a cause of death or only present at the time of the death. Such determinations are complex, as they can depend on the deceased’s medical history as well as substance levels that are influenced by the timing of bodily fluid retrieval. We relied exclusively on the pathologists’ determination of the presence and/or lethality of specific substances in each case rather than arriving at our own conclusions from review of raw toxicology data. We recorded the presence of all substances and whether they were determined by the pathologist to be a cause of death (i.e., at lethal levels). These included psychotropic medications, non-psychotropic medications, over the counter medications, alcohol, illicit substances, and poisons.

### Diagnosis

Using the same methodology which was previously published by our group (Schaffer et al. 2014), the presence of a diagnosis of BD was established based on information in the coroner’s investigation report. Diagnostic information was obtained by the coroner from a variety of sources, including medical records from the decedent’s physician(s), collateral information from family, police report of personal documents, and content of the suicide note that stated a diagnosis of BD or the presence of depressive symptoms in the absence of BD. This ‘unipolar depression’ group is comprised likely of individuals who fit into a variety of depressive conditions (Sinyor et al. 2015). Specific symptom criteria or psychological autopsies were not available. All suicide deaths with BD or unipolar depression were included in the analysis.

### Statistical analysis

Comparisons of subgroups were conducted by univariate analyses (*t*-tests or *χ* ^2^) of all demographic, clinical, stressor, and suicide-specific variables. In order to identify independent contribution to variance in the presence or absence of a self-poisoning method of suicide, multivariate logistic regression was completed, including all pre-death variables with a *p* < .1 identified in the univariate analyses.

Differences in the proportion of different medication classes or specific agents being present or lethal at the time of death were compared between the BD and non-BD as well as BD and unipolar depression self-poisoning groups using a series of *χ* ^2^ tests.

### Ethical approval and privacy

The OCC granted approval to this study and provided full access to their records for the purposes of completing this study. The study was approved by the Research Ethics Board at Sunnybrook Health Sciences Centre, Toronto, Canada. Strict privacy procedures utilized by the OCC were fully adhered to, with all extracted data maintained in an encrypted and de-identified format.

## Results

During the study period, 207 people with BD died by suicide, out of a total of 3319 suicide deaths. Among those with BD, 76 (36.7%) died by self-poisoning. There were also 585 self-poisoning suicide deaths in people that did not have BD. Table 1 characterizes 4 groups (BD self-poisoning, BD non-self-poisoning, non-BD self-poisoning, non-BD non-self-poisoning) with regards to demographics, other clinical history, recent stressors, and suicide details. There were 1764 people with unipolar depression who died by suicide, of which, 409 (23.2%) died by self-poisoning.

**Table 1:** Characteristics of bipolar disorder self-poisoning, bipolar disorder non-self poisoning, and non-bipolar disorder self-poisoning suicide deaths

Variable	Bipolar disorder suicide deaths by self-poisoning (*n* = 76)	Bipolar disorder suicide deaths by other methods (*n* = 131)	Non-bipolar disorder suicide deaths by self-poisoning (*n* = 585)	Non-bipolar disorder suicide deaths by other methods (*n* = 2527)	BD suicide deaths by self-poisoning vs. BD suicide deaths by other methods		BD suicide deaths by self-poisoning vs. non-BD suicide deaths by self-poisoning		BD suicide deaths by self-poisoning vs. non-BD suicide deaths by other methods	
					*X* or *t*-score	*p*-value	*X* or *t*-score	*p*-value	*X* or *t*-score	*p*-value
Demographics										
Sex (% male)	38.2	67.2	52.5	76.0	16.5	*<.0001*	5.73	.057	56.50	*<.0001*
Age (mean years, SD)	46.1, 11.3	42.6, 14.7	51.7, 16.0	46.3, 18.1	1.82	.071	−2.96	*.003*	−.084	*<.0001*
Age categories (%) (years)										
≤24	<*n* = 5	9.9	32.5	11.4	3.01	.222	11.84	*.003*	15.40	*<.0001*
25–64	92.1	84.0	76.6	71.7						
≥65	<*n* = 5	6.1	20.2	16.9						
Marital status (%)										
Married/common low	18.4	22.1	21.2	27.0	.733	.866	4.84	.184	7.10	.131
Divorced/separated/widowed	23.7	19.8	22.7	18.4						
Single/no status available	57.9	58.0	56.1	54.6						
Living circumstances (% with others)	48.7	54.2	45.8	58.0	.586	.444	.223	.637	2.63	.105
Other history										
Past suicide attempts (% yes)	63.2	40.5	42.6	22.0	9.92	.*002*	11.53	*.001*	70.13	*<.0001*
Psychiatry/emergency room visit in past week (%)	14.5	16.0	6.2	7.9	.089	.765	7.05	*.008*	4.33	*.037*
Comorbid substance abuse (% drug or alcohol)	34.2	22.9	27.9	18.1	3.12	.077	1.33	.249	12.61	*<.0001*
Comorbid medical condition (%)	40.8	29.0	53.8	28.5	3.00	.083	4.59	.*032*	5.40	.*020*
Recent stressors										
Any identified stressor (%)	36.8	41.2	51.5	52.3	.39	.535	5.74	*.017*	7.04	*.008*
Bereavement (%)	7.9	4.6	8.7	4.5	.97	.325	.058	.810	1.92	.166
Employment/Financial (%)	9.2	16.0	12.5	20.1	1.91	.167	.675	.411	5.48	.*019*
Interpersonal-conflict or relationship breakup (%)	21.1	22.1	20.9	23.6	.03	.855	.002	.968	.26	.608
Recent health/medical (%)	<*n* = 5	6.9	17.3	11.3	1.72	.190	10.95	.*001*	5.66	*.017*
Police/legal (%)	6.6	5.3	4.8	7.1	.13	.714	.456	.500	.03	.855
Suicide details										
Presence of a suicide note (%)	36.8	33.6	39.3	28.9	.23	.636	.173	.677	2.26	.133
Suicide at home (%)	75.0	61.1	72.5	61.2	4.17	*.041*	.216	.642	5.96	.*015*

Self-poisoning: includes drug or alcohol self-poisoning deaths

### BD self-poisoning vs. BD other methods

As shown in Table 1, those with BD who died by self-poisoning as compared to other methods were significantly more likely to be female, to have made a prior suicide attempt, and to have died at home. There were non-significant trends towards the BD self-poisoning group being older, and more likely to have comorbid substance abuse or comorbid medical condition.

Multivariate logistic regression (Table 2) found several variables to be independently associated with self-poisoning being the method of suicide among people with BD, including female sex, past suicide attempts, and comorbid substance abuse.

**Table 2:** Regression model of variables associated with self-poisoning among suicide decedents with bipolar disorder (*n* = 207)

Variable	Odds ratio	95% CI		*p*-value
		Lower	Upper	
Female sex	3.79	1.987	7.203	*<.0001*
Age	1.02	.995	1.044	.129
Past suicide attempts	2.25	1.209	4.191	*.011*
Comorbid substance abuse	2.44	1.200	4.955	*.014*
Comorbid medical condition	1.19	.598	2.381	.616

All pre-death variables with *p* < .1 in univariate analyses were included

An additional regression examining association and interaction of diagnosis (BD or non-BD) and sex with self-poisoning method was conducted

A non-significant interaction term (*p* = .083) was found

### BD self-poisoning vs. unipolar depression self-poisoning groups

The BD self-poisoning group compared to the unipolar depression self-poisoning group had a significantly higher proportion of past suicide attempt(s) (63.2 vs. 49.4%, *χ* ^2^ (1) = 4.87, *p* < .05), proportion of individuals with a psychiatry/ER visit(s) in the previous week (14.5 vs. 6.8%, *χ* ^2^ (1) = 5.04, *p* < .05), and proportion of adult age decedents (92.1 vs. 78.5%, *χ* ^2^ (1), 7.61, *p* < .05). In contrast, there was a significantly higher proportion of older adults (18.1 vs. 3.9%, *χ* ^2^ (1) = 9.60, *p* < .01), individuals with a recent medical health stressor (14.9 vs. 2.6%, *χ* ^2^ (1) = 8.56, *p* < .01), and individuals with any stressor present (51.8 vs. 36.8%, *χ* ^2^ (1) = 5.76, *p* < .05) among those with unipolar depression who died by self-poisoning relative to those with BD who died by self-poisoning. Welch–Satterthwaite *t* test indicated that those with unipolar depression who died by self-poisoning (mean age 50.9 years; SD = 15.4) were significantly older than those with BD who died by self-poisoning (mean age = 46.1 years; SD = 11.3) (*t* (132.59) = −3.09, *p* < .01).

### Lethal substances/medications in BD and non-BD self-poisoning groups

**Fig. 1:**
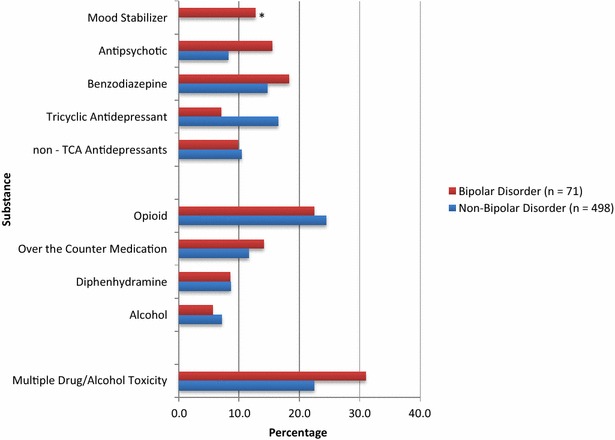
Lethal levels of substances present at suicide by self-poisoning among people with or without bipolar disorder. *p ≤ .0001. Any data point with *n* < 5 has been suppressed due to privacy limits. As such, values for Lithium (BD and Non-BD), Carbamazepine (BD and Non-BD), Valproate (BD and Non-BD) and Any Mood Stabilizer (Non-BD) have been suppressed. *Unknown multiple drug/alcohol toxicity* includes cases where multiple substances were present but the coroner was unable to specifically identify which substances were responsible for death. No bar was shown for suppressed data (cell size <5). Non-TCA Antidepressants include: SSRIs, SNRIs, NDRIs, MAOIs. Any Antidepressant refers to TCAs or the ‘non-TCA antidepressants’ (i.e. SSRIs, SNRIs, etc.). Total number of lethal substances not available for some cases as toxicology analysis was indeterminate; therefore number of lethal substances unknown for some. In some cases, typically where a number of substances were present in nonlethal levels, the pathologist concluded the cause of death was multiple drug toxicity without specifying which specific substances were responsible. This occurred in *n* = 21 BD cases and *n* = 129 non-BD cases. These suicides were included in the overall analysis but without a specific substance as the cause of death

The specific substances and classes of medications that were present at lethal levels at the time of self-poisoning suicide are shown in Fig. 1. In both the BD and non-BD groups, opioids were the most common class of lethal medication. There was a non-significant trend of higher likelihood of lethal levels of opioids among BD decedents (excluding *n* = 21 with unknown multiple drug toxicity) who had comorbid substance abuse compared to no such comorbidity (47.1 vs. 22.9%; *χ* ^*2*^ = 3.15, d*f* = 1, *p* = .076). There was no difference in identification of lethal opioids based on substance abuse comorbidity in the non-BD group (45.2 vs. 39.8%; *χ* ^*2*^ = 1.26, d*f* = 1, *p* = .26).

For the BD group, opioids were followed by benzodiazepines, antidepressants, and antipsychotics as the next most common classes of identified lethal medication taken. Nearly a third of the BD group had multiple drug/alcohol toxicity as the cause of death.

There were no significant differences in frequency of opioids, OTC medications, or alcohol between BD and non-BD groups. The mean number of lethal substances taken by people with BD who died by self-poisoning (1.76, SD = 1.2) was significantly higher than the number taken by people without BD (1.47, SD = 1.03) (*t* = 1.86, d*f* = 446, *p* = .064).

### Lethal substances/medications in BD and unipolar depression self-poisoning groups

A significantly higher proportion of lethal antipsychotics (21.2 vs. 8.8%, *χ* ^2^ (1) = 6.95, *p* < .01) and mood stabilizers (17.3 vs. .7%, Fisher’s Exact Test, *p* < .001) was noted among those with BD who died by self-poisoning relative to those with unipolar depression who died by self-poisoning. There was a significantly higher proportion of lethal TCA ingestion among those with unipolar depression who died by self-poisoning relative to those with BD who died by self-poisoning (23.4 vs. 9.6%, %, *χ* ^2^ (1) = 4.99, *p* < .05). The mean total number of lethal substances was not significantly different between those with BD who died by self-poisoning (mean = 1.76; SD = 1.23) and those with unipolar depression who died by self-poisoning (mean = 1.52; SD = 1.04) groups, *p* > .05.

### Substances/medications present in BD and non-BD self-poisoning groups

**Fig. 2:**
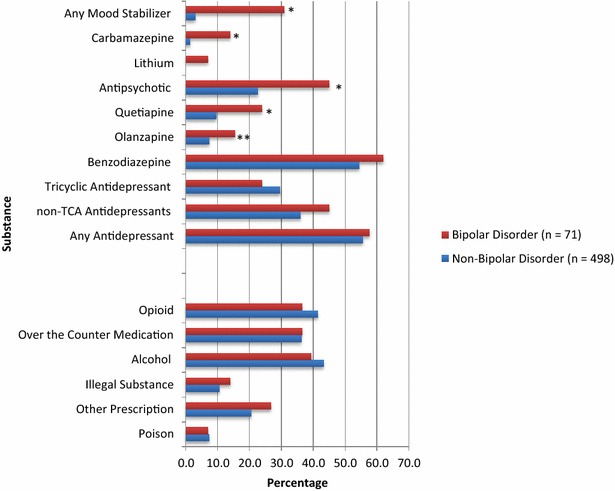
Substances present at any level at suicide by self-poisoning among people with or without bipolar disorder. *p ≤ .0001. **p = .022. Any data point with *n* < 5 has been suppressed due to privacy limits. As such, Valproate (BD and Non-BD) and Lithium (Non BD) have been suppressed. No bar was shown for suppressed data (cell size <5). Non-TCA Antidepressants include: SSRIs, SNRIs, NDRIs, MAOIs. Any antidepressant refers to TCAs or the ‘non-TCA antidepressants’ (i.e. SSRIs, SNRIs, etc.). Total number of lethal substances not available for some cases as toxicology analysis was indeterminate; therefore number of lethal substances unknown for some

Figure 2 displays the substances present at the time of death among BD and non-BD self-poisoning suicide decedents. For the BD group, benzodiazepines were the most common type of medication present at time of death (62%), followed closely by antidepressants (58%). Only 31% of those with BD had any type of traditional mood stabilizer detected, of which carbamazepine (14%) was the most common. Thirty-nine percent had detectable alcohol and had 14% had an illegal substance present. Thirty-seven percent of the BD group had some level of opioid present at the time of death. There was a non-significant trend of higher likelihood of opioids being present among BD decedents with comorbid substance abuse compared to no such comorbidity (52.2 vs. 29.2%; *χ* ^*2*^ = 3.56, d*f* = 1, *p* = .06). There was no such difference in opioid presence in the non-BD group (45.2 vs. 39.8%; *χ* ^*2*^ = 1.26, d*f* = 1, *p* = .26).

The presence of antidepressants and benzodiazepines were not significantly different between BD and non-BD groups, with only higher frequency of mood stabilizer (*χ* ^*2*^ = 79.98, d*f* = 1, *p* = < .0001) and antipsychotic use (*χ* ^*2*^ = 16.30, d*f* = 1, *p* = < .0001) among BD decedents differentiating the groups. There was a mean total of 4.6 (SD = 1.9, range 0–10) different substances present at time of death in the BD self-poisoning group, significantly higher than the mean total of 3.9 (SD = 2.1, range 0–12) in the non-BD self-poisoning group (*t* = *2.78,* d*f* = 567, *p* = .006).

### Antidepressants, mood stabilizers, and antipsychotics present in BD and non-BD self-poisoning groups

**Fig. 3:**
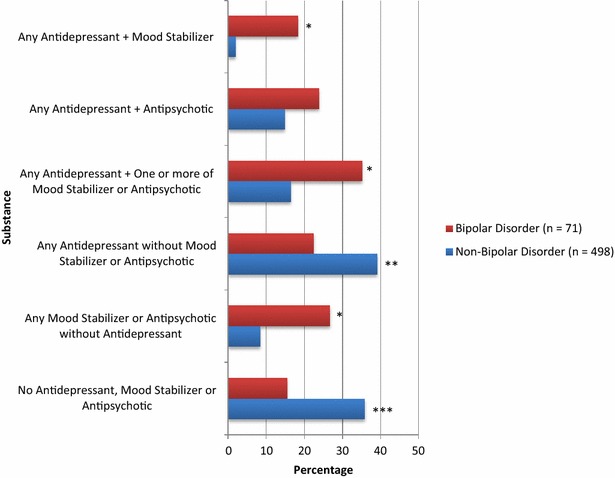
Antidepressant, mood stabilizer and antipsychotic medications present at self-poisoning suicide among people with or without bipolar disorder. *p ≤ .0001, **p = .007, ***p = .001

Figure 3 examines more closely the presence of antidepressants with or without concomitant mood stabilizers or antipsychotics. No antidepressant, mood stabilizer, or antipsychotic was present in 15.5% of the total BD group. Antidepressants were present in 58% of the BD group, of which 39% did not have at least one mood stabilizer or antipsychotic concomitantly present. As expected, differences in medications present between BD and non-BD groups were evident, with only the combination of antidepressant plus antipsychotic not being significantly different.

### Substances/medications present in BD and unipolar depression self-poisoning groups

A significantly higher proportion of antipsychotics (45.1 vs. 22.4%, *χ* ^2^ (1) = 15.63, *p* < .001) and mood stabilizers (31.0 vs. 2.8%, *χ* ^2^ (1) = 67.15, *p* < .001) was noted among those with BD who died by self-poisoning relative to those with unipolar depression who died by self-poisoning.

## Discussion

This large study of suicide deaths in BD found that self-poisoning accounted for 36.7% of all deaths, which is somewhat higher than other large studies (Chen et al. 2009; Hunt et al. 2006; Ösby et al. 2001), but within the published range from all reports (Schaffer et al. 2015b). Self-poisoning as the method of suicide for those with BD was significantly correlated with female sex, prior suicide attempts, and comorbid substance abuse. As far as we are aware, this is a new finding that requires replication from other sources, and if consistently reported would be an important aspect of understanding which subgroups within BD may be at particular risk of dying by self-poisoning as compared to other methods.

Within the BD group, females accounted for 61.8% of all self-poisoning suicides as compared to 32.8% by other methods, a significant difference on univariate analysis that was maintained in the regression model with an OR 3.79. These findings have relevance to suicide risk assessments which almost universally emphasize higher risk for suicide in men, but which should also consider that within the context of self-poisoning among people with BD, women account for more suicides than men, and self-poisoning is more predominantly used among women with BD.

We found that opioids were the most common type of identified lethal substance in both the BD and non-BD groups. While the literature is clear that opioids are a common source of lethal self-poisoning, we were surprised for this to also be the case in people with BD. This may be influenced by our finding of a non-significant trend towards more frequent opioid lethality among BD decedents with comorbid substance abuse, which was not the case in the non-BD group. How the opioids were obtained was not known to us, so we cannot speculate on the proportion of prescribed or non-prescribed sources, but growing evidence points to extensive access to prescribed opioids among the population in general (Tetrault and Butner 2015), including those at risk of suicide (Ekholm et al. 2014; Madadi et al. 2013; Madadi and Persaud 2014; West et al. 2015). Patients with BD are known to have higher than expected rates of chronic pain (Stubbs et al. 2015), and pain can certainly precipitate suicidal behavior; however, the possibility of drug misuse in the context of a substance use disorder or management of psychological pain is clearly present. The significant lethality associated with opioid self-poisoning further increases the risk of opioid use in those at elevated risk of suicide and suggests extra vigilance is required for this population to ensure that appropriate means restriction measures are in place.

Benzodiazepines and antidepressants were the next most common classes of lethal substances used among people with BD and were also the most common classes present in the body at time of death. This indirectly suggests that frequency of use was a larger factor than specific lethality risk of the compounds. Coroner data did not permit an examination of details of pharmacotherapy such as indications for use, duration of therapy, or dosing and prescribing patterns of medications, and therefore, it is difficult to evaluate the appropriateness of pharmacotherapy. Nonetheless, the greater frequency benzodiazepine and antidepressant use as compared to lithium or anticonvulsants suggests that a large number of patients were not receiving BD guideline-concordant care at the time of death. Furthermore, we found that 39% of those with BD who had an antidepressant present at the time of death did not have any concomitant mood stabilizer or antipsychotic present. Nearly 1/6th of BD decedents did not have any antidepressant, mood stabilizer, or atypical antipsychotic present. To what degree these findings relate to issues of adherence, physician selection of therapy, or other factors is not known. The lack of a living control group negates any possible discussion of risk of suicide, however identifying what appears to be low rates of guideline-concordant care (Goodwin and Consensus Group of the British Association for 2009; Grunze et al. 2010; Yatham et al. 2013) is very concerning.

We can speculate that the relationship between antidepressant/benzodiazepine use and suicide in BD may be partially mediated through high rates of comorbid anxiety (Schaffer et al. 2007; Schaffer et al. 2012), treatment resistance (Yatham et al. 2005), illness severity (Marangell et al. 2008), insomnia (Chung et al. 2015; Pompili et al. 2013b) and possible induction of mixed states or rapid cycling (Pacchiarotti et al. 2013; Viktorin et al. 2014). The findings may also be a function of higher prescriptions rates for antidepressants and benzodiazepines among women with BD compared to men with BD (Schaffer et al. 2006; Weinstock et al. 2014). At a minimum, clinicians and researchers should be aware of the pattern of pharmacotherapy use at time of self-poisoning suicide death, which deserves further clinical and research attention.

Most of the extant literature on BD pharmacotherapy and suicide has focused on lithium and anticonvulsants (Cipriani et al. 2013; Oquendo et al. 2011; Schaffer et al. 2015b). We found that only 7% of people with BD who died by suicide had any lithium present at the time of death, and the number of those with lethal levels was below the threshold for reporting based on privacy restrictions (i.e., *n* = 5). In contrast, anticonvulsants were present at any level in 21% of BD suicide deaths, most commonly being carbamazepine (14%), with the other anticonvulsants below the privacy threshold. The high number of deaths in which carbamazepine was present was an unexpected finding, especially given the declining use in clinical practice for BD, which is currently estimated at 3% of BD patients (Mauer et al. 2014). There is a modest literature suggesting higher rates of suicide attempts during treatment with carbamazepine monotherapy compared to lithium monotherapy (Cipriani et al. 2013; Goodwin et al. 2003), but other factors such as relative toxicity in overdose (Spiller et al. 1990) and profile of patients who are prescribed carbamazepine (Leon et al. 2012) may also be at play.

Presumably reflecting the changing practices in management of BD, antipsychotics were more often present than traditional mood stabilizers, with quetiapine and olanzapine being the two antipsychotics that surpassed the privacy threshold. While our data could not examine relative anti-suicide effects, it is noteworthy that there is only very sparse data on the impact of antipsychotic medications on suicide risk in patients with BD (Koek et al. 2012; Yerevanian et al. 2007).

The significantly higher proportion of past suicide attempts and psychiatry/ER visits in the prior week among the BD self-poisoning group relative to unipolar depression self-poisoning group is consistent with previous epidemiologic studies showing more frequent mental health care contacts among BD and schizophrenia suicide decedents (Schaffer et al. 2016). With regard to lethal and present medications, the patterns of differences in proportion of medications between BD and unipolar self-poisoning groups are essentially reflective of more commonly prescribed medications among these populations.

There are a number of limitations that should be considered when interpreting the results of this paper. First, the diagnosis of BD was based on findings from coroner investigations that rely on multiple sources, but do not include a structured diagnostic assessment, and misdiagnosis is therefore likely to have occurred in at least some cases, most likely as false negatives. However, the BD suicide group accounted for 6.2% of all suicide deaths, which is very much in keeping with prior studies on the proportion of BD decedents in large suicide samples (Karch et al. 2006; Schaffer et al. 2014; Takizawa 2012), and there was 10-fold greater use of mood stabilizers in the BD group and small percentage (3%) in the non-BD group. Second, the lack of a living control group limits any conclusions related to risk impact of the pharmacotherapies identified or of correlates of method type, as potential causality could not be examined. Third, the comparison group of non-BD suicide decedents is in fact a mix of other diagnoses or no diagnosis at all. We chose to include a broad comparison group in order to allow for basic comparisons to be made for better characterization of the BD subgroup. Furthermore, while we identified differences between BD and unipolar depression groups, uncertainty regarding diagnostic precision may limit the interpretation of results. Fourth, the data on correlates of self-poisoning as the method of suicide death were limited by the variables available in coroner data, which did not include many illness related factors that are known to influence suicide risk (Schaffer et al. 2015a), and could not differentiate medications taken as part of treatment as opposed to only during the act of self-poisoning. Furthermore, it is difficult to know whether the clinical differences impact a greater propensity to using self-poisoning as the method of attempt, greater access to large amounts of substances, higher lethality as a result of underlying medical vulnerability, or whether clinical aspects of the diagnosis were a key aspect of the suicide death. Fifth, coroner’s toxicology results necessarily examine only substances present at the moment of death, and do not include information on doses of prescribed medications or provide information as to the source or intended use of the compound. This is especially relevant for medication classes such as opioids, which have broad-spectrum analgesic effects, but are also subject to abuse and dependence. Furthermore, this study could not answer important questions about recent treatment such as how many people discontinued a mood stabilizer days or weeks prior to their deaths.

This study highlights the specific characteristics of people with BD who die by self-poisoning, a subgroup that accounts for over a third of all BD suicide deaths. Female sex, past suicide attempts, and comorbid substance abuse each significantly increased the likelihood of self-poisoning being the method of suicide among people with bipolar disorder (BD), and clinicians should maintain a high index of suspicion for self-poisoning suicide in these patients.

Additional new findings related to the types of substances present and lethal at the time of death are informative at both a clinical and policy level, and if replicated in other populations suggest the need for heightened attention to issues of medication access and choices for those at greater risk of suicide.
